# Incidence and mortality of female breast cancer in the Asia-Pacific region

**DOI:** 10.7497/j.issn.2095-3941.2014.02.005

**Published:** 2014-06

**Authors:** Danny R. Youlden, Susanna M. Cramb, Cheng Har Yip, Peter D. Baade

**Affiliations:** ^1^Cancer Council Queensland, Brisbane 4006, Australia; ^2^School of Mathematical Sciences, Queensland University of Technology, Brisbane 4000, Australia; ^3^Faculty of Medicine, University of Malaya, Kuala Lumpur 50603, Malaysia; ^4^Griffith Health Institute, Griffith University, Gold Coast 4222, Australia; ^5^School of Public Health and Social Work, Queensland University of Technology, Brisbane 4000, Australia

**Keywords:** Asia-Pacific region, female breast cancer, epidemiology, incidence, mortality

## Abstract

**Objective:**

To provide an overview of the incidence and mortality of female breast cancer for countries in the Asia-Pacific region.

**Methods:**

Statistical information about breast cancer was obtained from publicly available cancer registry and mortality databases (such as GLOBOCAN), and supplemented with data requested from individual cancer registries. Rates were directly age-standardised to the Segi World Standard population and trends were analysed using joinpoint models.

**Results:**

Breast cancer was the most common type of cancer among females in the region, accounting for 18% of all cases in 2012, and was the fourth most common cause of cancer-related deaths (9%). Although incidence rates remain much higher in New Zealand and Australia, rapid rises in recent years were observed in several Asian countries. Large increases in breast cancer mortality rates also occurred in many areas, particularly Malaysia and Thailand, in contrast to stabilising trends in Hong Kong and Singapore, while decreases have been recorded in Australia and New Zealand. Mortality trends tended to be more favourable for women aged under 50 compared to those who were 50 years or older.

**Conclusion:**

It is anticipated that incidence rates of breast cancer in developing countries throughout the Asia-Pacific region will continue to increase. Early detection and access to optimal treatment are the keys to reducing breast cancer-related mortality, but cultural and economic obstacles persist. Consequently, the challenge is to customise breast cancer control initiatives to the particular needs of each country to ensure the best possible outcomes.

## Introduction

Until recently, information on the epidemiology of female breast cancer was mainly gleaned from studies conducted in the Western world^1^^,^^2^. In 1990, it was estimated that 59% of breast cancer cases occurred in more developed countries (defined as North America, Europe, Australia, New Zealand and Japan), although these areas accounted for less than a quarter of the global female population at the time^3^. The situation changed considerably over the next two decades; by 2008, the total number of new diagnoses were evenly divided between more developed and less developed countries^4^^,^^5^, and by 2012 it was estimated that the majority (53%) of cases of female breast cancer were occurring in less developed countries^6^. While incidence rates still remain much higher in more developed countries, this shift in the global distribution of cases highlights that breast cancer is continuing to emerge as a major health issue for women in Asia, Africa and South America.

The Asia-Pacific region includes Eastern and South-Eastern Asia as well as Oceania (see **Table 1** for a full list of the countries included)^6^. It comprises a diverse mix of geography, cultures and economies^1^, and is home to almost a third (32%) of the global female population^7^. This region is of specific interest because the annual increases in female breast cancer incidence since 1990 in some parts of the Asia-Pacific have been reported to be up to eight times higher than the world average^8^^,^^9^.

**Table 1:** Estimated breast cancer incidence and mortality by country, Asia-Pacific region, 2012

Region/country	Incidence			Mortality		MR:IR	Data availability and methods
	Cases	ASR		Cases	ASR		
World	1,676,633	43.3		521,817	12.9	0.30	
Asia-Pacific	403,876	29.6		115,863	8.1	0.27	
Eastern Asia	277,054	27.0		68,531	6.1	0.23	
China	187,213	22.1		47,984	7.3	0.33	C4
Japan	55,710	51.5		13,801	9.8	0.19	B1
Mongolia	125	9.4		50	4.2	0.45	D5
North Korea	5,707	36.8		2,340	14.3	0.39	G6
South Korea	17,140	52.1		2,274	6.1	0.12	A2
South-Eastern Asia	107,545	34.8		43,003	14.1	0.41	
Brunei	83	48.6		18	11.3	0.23	F5
Cambodia	1,255	19.3		585	9.3	0.48	G6
Indonesia	48,998	40.3		19,750	16.6	0.41	F6
Laos	472	19.0		222	9.3	0.49	G6
Malaysia	5,410	38.7		2,572	18.9	0.49	C2
Myanmar	5,648	22.1		2,792	11.3	0.51	G6
Philippines	18,327	47.0		6,621	17.8	0.38	C2
Singapore	2,524	65.7		628	15.5	0.24	A1
Thailand	13,653	29.3		5,092	11.0	0.38	B3
Timor-Leste	108	32.6		52	16.4	0.50	G6
Viet Nam	11,067	23.0		4,671	9.9	0.43	E4
Oceania	19,277	79.2		4,329	15.6	0.20	
Australia	14,710	86.0		2,968	14.0	0.16	A1
New Zealand	2,840	85.0		652	17.1	0.20	A1
Melanesia	1,376	41.0		633	19.7	0.48	
Fiji	277	65.0		114	28.4	0.44	D1
New Caledonia	129	87.6		35	23.4	0.27	D5
Papua New Guinea	848	33.7		428	17.7	0.53	G6
Solomon Islands	92	47.6		44	24.4	0.51	G6
Vanuatu	30	31.8		12	13.4	0.42	D6
Micronesia	128	48.8		27	10.4	0.21	
Guam	51	49.4		11	10.2	0.21	D6
Polynesia	223	68.9		49	15.4	0.22	
French Polynesia	135	92.2		29	20.4	0.22	D5
Samoa	18	23.2		5	5.6	0.24	D6

Abbreviations: ASR, Age standardised rate/100,000 population; MR:IR, mortality to incidence rate ratio. Notes: Data availability and methods coded as follows: A, High quality national data or high quality regional (coverage greater than 50%); B, High quality regional (coverage between 10% and 50%); C, High quality regional (coverage lower than 10%); D, National data (rates); E, Regional data (rates); F, Frequency data; G, No data; 1, Rates projected to 2012; 2, Most recent rates applied to 2012 population; 3, Estimated as the weighted average of regional rates; 4, Estimated from national incidence estimates by modelling, using country-specific survival; 5, Estimated from national incidence estimates using modelled survival; 6, The rates are those of neighbouring countries or registries in the same area. Data source: GLOBOCAN^6^.

The purpose of this study was to describe and compare the latest available incidence and mortality data on female breast cancer for countries within the Asia-Pacific region. This is important in terms of monitoring changes in the burden of breast cancer over time and to allow some degree of benchmarking between countries, thus identifying where action is most required to prevent breast cancer and to improve outcomes for women diagnosed with breast cancer.

## Materials and methods

### Data

Incidence and mortality estimates for the year 2012 were extracted from the GLOBOCAN database compiled by the International Agency for Research on Cancer (IARC)^6^. The latest estimates available by age at diagnosis (under 50 *vs*. 50 years and older) were for 2008^10^. Data on incidence by stage at diagnosis were sourced from individual publications. Only cases of primary invasive breast cancer (ICD-10/ICD-O-3: C50 or ICD-9: B113) were included, unless otherwise specified.

Since GLOBOCAN data are for a single year, we also obtained longitudinal incidence data for trend analyses from IARC^11^ and/or individual cancer registries^12^^-^^16^ where available. Potential cancer registries were identified through online searches. Data was either downloaded directly from the website, or Registrars were contacted when data was unavailable online. **Table 2** lists the countries for which incidence trend data were obtained.

**Table 2:** Annual percent change (APC) in the incidence rates of breast cancer among females by country/registry area and broad age group, 1980–2011

Country	Trend 1				Trend 2				Trend 3				Trend 4			
	Year	APC	(95% CI)		Year	APC	(95% CI)		Year	APC	(95% CI)		Year		APC	(95% CI)
All ages																
Australia	1982-1995	+3.1*	(+2.6, +3.6)		1995-2009	+0.2	(–0.2, +0.5)									
China (Shanghai)	1980-2005	+3.4*	(+3.0, +3.8)													
Hong Kong	1983-1993	+0.7	(–0.4, +1.9)		1993-2011	+2.4*	(+2.1, +2.7)									
Japan	1980-1986	+5.3*	(+3.5, +7.2)		1986-1999	+2.1*	(+1.6, +2.6)		1999-2008	+6.1*	(+5.5, +6.7)					
New Zealand	1980-1999	+2.0*	(+1.5, +2.4)		1999-2009	–0.2	(–1.0, +0.7)									
Philippines	1983-2002	+0.6*	(+0.2, +1.0)													
Singapore	1980-2002	+3.8*	(+3.4, +4.1)													
Thailand	1983-2009	+3.9*	(+3.3, +4.5)													
<50 years																
Australia	1982-1993	+1.8*	(+1.2, +2.3)		1993-2009	+0.2	(-0.0, +0.5)									
China (Shanghai)	1988-1995	–0.7	(–3.3, +2.0)		1995-2002	+3.6*	(+1.3, +5.9)									
Hong Kong	1983-2011	+1.9*	(+1.7, +2.2)													
Japan	1980-1987	+5.3*	(+3.8, +6.8)		1987-2001	+1.8*	(+1.3, +2.3)		2001-2008	+6.3*	(+5.2, +7.4)					
New Zealand	1980-2009	+1.0*	(+0.7, +1.3)													
Philippines	1983-1989	+1.5	(–1.8, +4.9)		1989-1992	–4.4	(–20.2, +14.5)		1992-1995	+7.1	(–8.8, +25.8)		1995-2002	–2.1*		(–4.0, –0.1)
Singapore	1980-2002	+2.8*	(+2.3, +3.3)													
Thailand	1983-2002	+4.4*	(+2.3, +6.4)													
50+ years																
Australia	1982-1997	+3.4*	(+2.9, +3.9)		1997-2009	–0.2	(–0.8, +0.4)									
China (Shanghai)	1988-1997	+2.7*	(+0.9, +4.5)		1997-2002	+7.9*	(+3.8, +12.1)									
Hong Kong	1983-1993	–0.2	(–1.4, +1.1)		1993-2011	+2.7*	(+2.4, +3.1)									
Japan	1980-1986	+4.8*	(+2.7, +6.9)		1986-1998	+2.4*	(+1.7, +3.0)		1998-2008	+6.4*	(+5.9, +7.0)					
New Zealand	1980-2000	+2.3*	(+1.9, +2.8)		2000-2009	–1.0	(–2.1, +0.1)									
Philippines	1983-2002	+0.8*	(+0.4, +1.3)													
Singapore	1980-2002	+4.5*	(+4.1, +5.0)													
Thailand	1983-1997	+2.0*	(+0.4, +3.5)		1997-2002	+9.6*	(+4.2, +15.2)									

Notes: * Indicates that the APC is significantly different from zero at the *P*=0.05 level. CI=confidence interval. Trends calculated using Joinpoint regression. Rates were directly age-standardised to the World Standard population^17^. Data sources: Australia—Australian Institute of Health and Welfare^15^; China—Shanghai Cancer Registry^11^; Hong Kong—Hong Kong Cancer Registry^13^; Japan—Center for Cancer Control and Information Services^14^; New Zealand—Ministry of Health^16^; Philippines—Manila Cancer Registry^11^; Singapore—National Cancer Centre Singapore (residents of Chinese ethnicity only)^11^; Thailand—Chiang Mai Cancer Registry^11^^,^^12^.

Mortality trend analyses used data from the World Health Organisation (WHO) Mortality Database^18^, which contains cause of death by age, sex and year as reported by individual countries. Countries within the Asia-Pacific region were included if they were in the WHO Mortality Database, had at least ten years of data available during 1980-2011, and an annual average of at least 100 deaths/year due to breast cancer during the most recent five years. Eligible countries for the mortality trend analyses are listed in **Table 3**. The criteria resulted in the exclusion of Brunei, Fiji, Kiribati, and Papua New Guinea. Several other countries, including Indonesia, North Korea, Viet Nam and Cambodia were not listed in the WHO Mortality Database.

**Table 3:** Annual percent change (APC) in the mortality rates of breast cancer among females by country and broad age group, 1980-2011

Country	Trend 1				Trend 2				Trend 3				Trend 4		
	Year	APC	(95% CI)		Year	APC	(95% CI)		Year	APC	(95% CI)		Year	APC	(95% CI)
All ages															
Australia	1980-1985	+1.6	(–0.2, +3.3)		1985-1994	–0.4	(–1.2, +0.3)		1994-2000	–3.2*	(–4.7, –1.7)		2000-2011	–1.7*	(–2.2, –1.3)
China	1987-1995	+0.4	(–0.7, +1.5)		1995-2000	+4.1*	(+2.1, +6.2)								
Hong Kong	1980-2011	–0.1	(–0.3, +0.2)												
Japan	1980-1990	+1.5*	(+1.0, +1.9)		1990-1997	+3.3*	(+2.4, +4.2)		1997-2011	+1.1*	(+0.9, +1.3)				
Malaysia	1997-2008	+5.9*	(+5.4, +6.4)												
New Zealand	1980-1989	+0.4	(–0.9, +1.8)		1989-2009	–2.1*	(–2.4, –1.7)								
Philippines	1992-2008	+3.5*	(+2.9, +4.1)												
Singapore	1980-2011	+0.3	(–0.1, +0.6)												
South Korea	1985-1994	+5.5*	(+4.0, +7.1)		1994-2011	+2.1*	(+1.7, +2.5)								
Thailand	1980-1985	–9.4	(–18.0, +0.1)		1985-1997	+5.8*	(+3.0, +8.7)		1997-2000	+28.2*	(+1.6, +61.8)		2000-2006	+6.8*	(+3.5, +10.2)
<50 years															
Australia	1980-1991	+0.7	(–0.5, +2.0)		1991-2011	–3.1*	(–3.6, –2.6)								
China	1987-2000	+3.3*	(+2.7, +3.9)												
Hong Kong	1980-2000	+0.6	(–0.2, +1.5)		2000-2011	–2.6*	(–4.1, –1.0)								
Japan	1980-1998	+2.2*	(+2.0, +2.5)		1998-2011	–1.3*	(–1.7, –0.9)								
Malaysia	1997-2008	+4.0*	(+3.3, +4.7)												
New Zealand	1980-1996	–0.5	(–1.6, +0.6)		1996-2009	–3.0*	(–4.4, –1.6)								
Philippines	1992-2008	+2.6*	(+1.8, +3.4)												
Singapore	1980-2011	–1.5*	(–2.1, –0.8)												
South Korea	1985-1993	+3.9*	(+1.7, +6.0)		1993-2011	+1.0*	(+0.6, +1.5)								
Thailand	1980-1986	–6.6	(–14.8, +2.4)		1986-1997	+7.5*	(+3.6, +11.6)		1997-2000	+24.7	(–4.7, +63.1)		2000-2006	+5.7*	(+1.7, +9.8)
50+ years															
Australia	1980-1994	+0.1	(–0.3, +0.6)		1994-1998	–4.1	(–8.0, +0.0)		1998-2011	–1.6*	(–2.0, –1.2)				
China	1987-1995	–0.4	(–1.6, +0.8)		1995-2000	+3.9*	(+1.7, +6.2)								
Hong Kong	1980-2011	+0.1	(–0.2, +0.4)												
Japan	1980-1990	+1.2*	(+0.7, +1.7)		1990-1997	+3.6*	(+2.6, +4.6)		1997-2011	+2.0*	(+1.8, +2.3)				
Malaysia	1997-2008	+7.0*	(+6.1, +7.8)												
New Zealand	1980-1989	+0.4	(–0.9, +1.6)		1989-2009	–2.0*	(–2.3, –1.6)								
Philippines	1992-2008	+3.8*	(+3.2, +4.5)												
Singapore	1980-2011	+0.8*	(+0.5, +1.2)												
South Korea	1985-1995	+6.8*	(+5.0, +8.5)		1995-2011	+2.7*	(+2.2, +3.2)								
Thailand	1980-1985	–9.8	(–20.0, +1.6)		1985-1997	+5.1*	(+1.7, +8.7)		1997-2000	+30.3	(–2.7,+74.3)		2000-2006	+7.4*	(+3.4,+11.6)

Notes: * Indicates the APC is significantly different from zero at the *P*=0.05 level. CI, confidence interval. Trends calculated using Joinpoint regression. Rates were directly age-standardised to the World Standard population^17^. Data source: World Health Organization^18.^ Population data for the Philippines and Thailand were obtained from the United Nations^7^.

### Statistical analysis

All incidence and mortality rates were directly age-standardised to the Segi World Standard population^17^. Rates were expressed per 100,000 female population, and were estimated for all ages combined, as well as being stratified into two broad age groups that approximate pre-menopausal (under 50 years old at diagnosis) and post-menopausal (50 years and over) women. Calculations were performed in Stata v12.0 (StataCorp, Texas, United States) and maps were generated in Manifold System v8.0 (Manifold Software Limited, Wanchai, Hong Kong).

Recent survival estimates based on a consistent methodology were not available for most countries in the Asia-Pacific region. Instead, the mortality to incidence rate ratio (MR:IR) was used to provide an approximation for the prospects of survival in each country. The MR:IR has a range between 0 and 1; lower values of MR:IR (closer to zero) denote higher rates of survival, while higher values of MR:IR (closer to one) indicate poorer survival.

Trends in incidence and mortality rates were analysed using the Joinpoint regression program v4.0.4 (National Cancer Institute, Bethesda). This method evaluates changing linear trends across consecutive time periods^19^. A ‘joinpoint’ occurs when the linear trend changes significantly in either direction or magnitude. All of the models were run under the same specifications, namely that a minimum of 6 years was required between a joinpoint and either end of the data series or at least 4 years of data between joinpoints, with a maximum of three joinpoints allowed. Trends were reported in terms of the annual percent change (APC).

## Results

### Incidence rates

**Figure 1:**
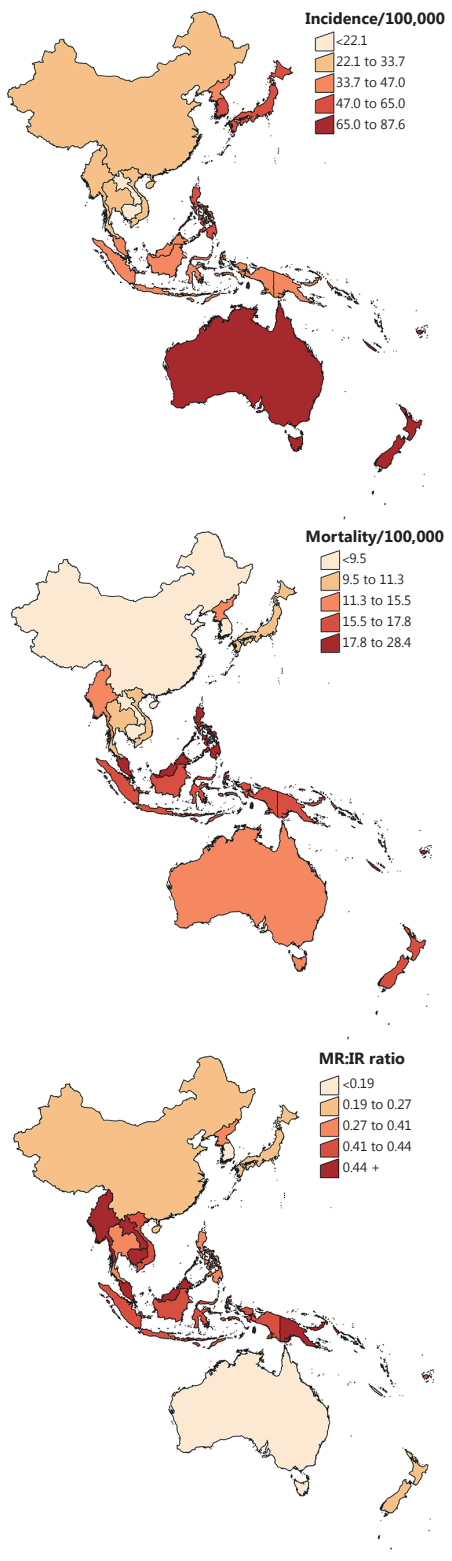
Breast cancer incidence, mortality and mortality rate: incidence rate ratio (MR:IR) for Asia-Pacific countries, 2012. Notes: Rates were age-standardised to the World Standard Population^17^ and expressed per 100,000 females. All categories were based on quintiles. Data source: GLOBOCAN^6^.

It was estimated that almost 1.7 million cases of female breast cancer were diagnosed worldwide during 2012, corresponding to a rate of 43 per 100,000 (**Table 1**). Close to a quarter (24%) of all breast cancers were diagnosed within the Asia-Pacific region (approximately 404,000 cases at a rate of 30 per 100,000), with the greatest number of those occurring in China (46%), Japan (14%), and Indonesia (12%). Incidence rates varied by around 10-fold across the region, ranging from an estimate of 9 per 100,000 in Mongolia up to 88 per 100,000 in New Caledonia and 92 per 100,000 in French Polynesia (**Table 1** and **Figure 1**). Australia (86 per 100,000) and New Zealand (85 per 100,000) also had much higher incidence rates than any of the other major countries in the region. The highest incidence of breast cancer for Eastern Asia occurred in Japan and South Korea (both 52 per 100,000) and for South-Eastern Asia the highest rate was in Singapore (65 per 100,000).

Breast cancer was the most common type of cancer among females in the Asia-Pacific, accounting for 18% of all cancer diagnoses. It also ranked first for females in the majority of countries within the region for which estimates were available (19 out of 26), except for Cambodia and Papua New Guinea where there were more cervical cancers, China and North Korea where there were a higher number of lung cancer cases, South Korea where breast cancer was second behind thyroid cancer, Laos where it ranked second after liver cancer and Mongolia where breast cancer was the fifth most common type of cancer diagnosed for females.

### Incidence by age

Globally, one in three women (33%) diagnosed with breast cancer were estimated to be aged under 50 at the time of diagnosis during 2008, compared to 42% throughout the Asia-Pacific region and 47% within the subregion of South-Eastern Asia. The proportion of female breast cancers that were diagnosed among women under 50 years of age ranged from 21% in Australia to 55% in South Korea and Laos and 58% in Vanuatu and Papua New Guinea (data not shown).

**Figure 2:**
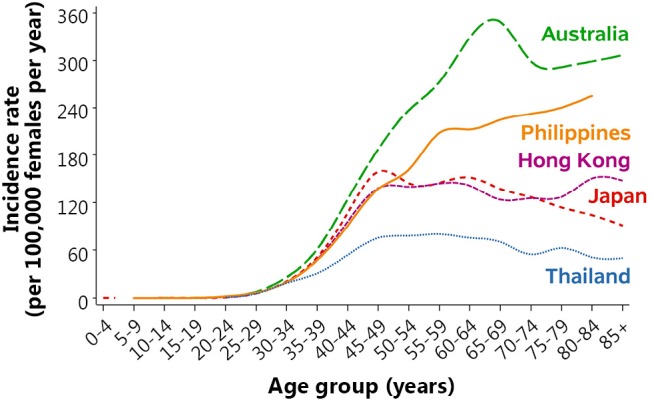
Age-specific incidence rates of breast cancer among selected countries, 2004-2008. Notes: Data was for 2004-2008 except for Philippines (2004-2007) and Thailand (2003-2007). Data for Philippines available to ages 80+. Data sources: Australia—Australian Institute of Health and Welfare^15^; Hong Kong—Hong Kong Cancer Registry^13^; Japan—Center for Cancer Control and Information Services^14^; Philippines (Manila) and Thailand (Chiang Mai)—International Association of Cancer Registries^20^.

A peak in the age-specific incidence rates occurred in Australia for women 50-69 years old (**Figure 2**), coinciding with the target age range for screening. Incidence rates in the Philippines continued to increase with advancing age. In contrast, age-specific incidence in Hong Kong, Japan and Thailand either plateaued or began to decrease for women aged 50 years and older.

### Incidence by stage

The stage at which breast cancer was diagnosed varied greatly throughout the Asia-Pacific region. More than half of the breast cancers in New South Wales, Australia (51%)^21^ and South Korea (56%)^22^ were detected at an early (localised) stage. Chiang Mai in Thailand had 21% diagnosed when localised (although a further 52% were diagnosed at ‘locally advanced’—a category not defined by other regions)^23^. Under the alternate staging system of categories I to IV, Japan had a high proportion of Stage I diagnoses (47% when excluding Stage 0)^24^, as did the state of Queensland in Australia (48%)^25^, while Singapore, China (Beijing) and Hong Kong had lower proportions ranging from 27% to 31%^26^^-^^28^. Malaysia had only 14% of breast cancers diagnosed at Stage I, but the unstaged category was very large (36%)^29^.

Even within countries there were often substantial disparities in stage at diagnosis between regions and/or ethnic groups. In New Zealand, Pacific Islanders tended to have fewer localised breast cancers diagnosed and a higher proportion of distant cancers, particularly when compared with women who were neither Maori nor Pacific Islanders^30^. Within China, women living in Beijing had a higher percentage of breast cancers detected at stage I (27%) and fewer stage IV cancers (0.3%) than other regions, particularly Sichuan (stage I: 3%, stage IV: 5%)^27^.

### Incidence trends

**Figure 3:**
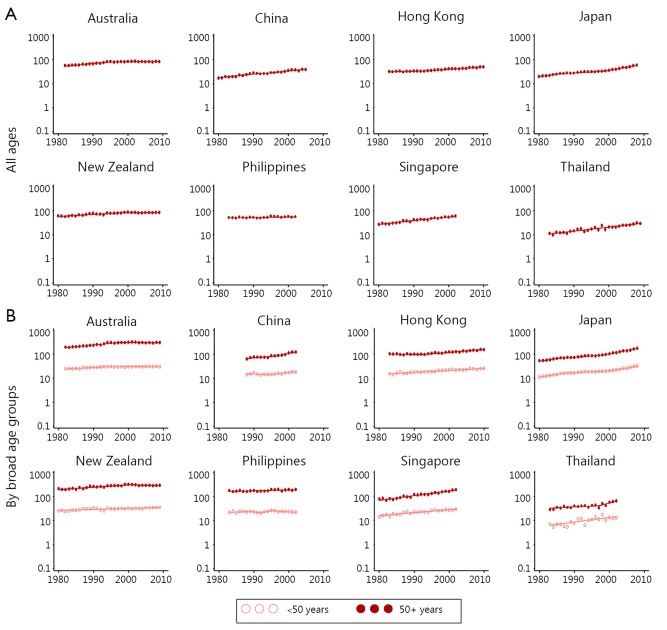
Breast cancer incidence rate trends for selected Asia-Pacific countries, 1980–2011. Notes: Y-axis is shown on a log scale. Rates were age-standardised to the World Standard Population^17^, and expressed per 100,000 female population. Years available differ by country, and sometimes by age groups. Singapore data was only available for residents of Chinese ethnicity. Data sources: Australia—Australian Institute of Health and Welfare^15^; China—Shanghai Cancer Registry^11^; Hong Kong—Hong Kong Cancer Registry^13^; Japan—Center for Cancer Control and Information Services^14^; New Zealand—Ministry of Health^16^; Philippines—Manila Cancer Registry^11^; Singapore—National Cancer Centre Singapore^11^; Thailand—Chiang Mai Cancer Registry^11^^,^^12^.

Significant increases in breast cancer incidence in recent years were observed in several Asian countries (**Table 2** and **Figure 3**), with incidence rates increasing by 3% to 4% per year in China (Shanghai), Singapore and Thailand. The largest rise was reported in Japan, where significant increases from 1980 onwards culminated in an average increase of 6% per year between 1999-2008. A different pattern was seen for both Australia and New Zealand, where incidence rates increased until the mid to late 1990s, but have since stabilised.

Similar incidence rate trends were found in both the under 50 and 50 and over age groups for female breast cancer within Australia and Japan. However, rates were increasing more rapidly for older women in China (Shanghai), Hong Kong, Singapore and Thailand, while in New Zealand the trend was increasing for those aged under 50 compared to some evidence of a possible decrease in incidence over the last decade for women aged 50 years and over.

### Mortality rates

About 522,000 females (13 per 100,000 population) were estimated to have died from breast cancer globally during 2012, including almost 116,000 deaths (22%) throughout the Asia-Pacific region at a rate of 8 per 100,000 (**Table 1**). China accounted for 41% of female breast cancer deaths within the region, followed by Indonesia (17%) and Japan (12%). Mortality rates by subregion varied from 6 per 100,000 in Eastern Asia to 14 per 100,000 in South-Eastern Asia and 16 per 100,000 in Oceania. Fiji was reported as having the highest mortality rate for female breast cancer in the Asia-Pacific (28 per 100,000) followed by the Solomon Islands (24 per 100,000 population) and New Caledonia (23 per 100,000), although the absolute number of deaths due to breast cancer in each of these countries was small (**Table 1** and **Figure 1**).

Breast cancer was estimated to account for 9% of cancer-related deaths among females in the Asia-Pacific region overall, ranking fourth behind lung, liver and stomach cancers. However, it was the leading cause of cancer-related deaths for females in several countries including Fiji, the Solomon Islands (both 27% of all cancer-related deaths), Malaysia (25%), the Philippines (23%), Indonesia (22%), New Caledonia, Vanuatu (both 21%), Singapore (20%) and Samoa (13%) and was the second most frequent in Guam (19%), French Polynesia (18%), Brunei (17%), Australia, New Zealand (both 16%), Papua New Guinea, Timor-Leste (both 15%) and North Korea (12%).

### Mortality by age

There were large differences across the Asia-Pacific region in the proportion of breast cancer deaths that were estimated to have occurred before 50 years of age during 2008. The regional average was 29% compared to 24% worldwide, but this varied from 17% in the subregion of Oceania up to 34% in South-Eastern Asia. The country-specific proportion of breast cancer mortality among women under 50 years old at the time of death was lowest in Japan and Australia (both 13%) and highest in Mongolia (39%) and Papua New Guinea (43%) (data not shown).

### Mortality trends

**Figure 4:**
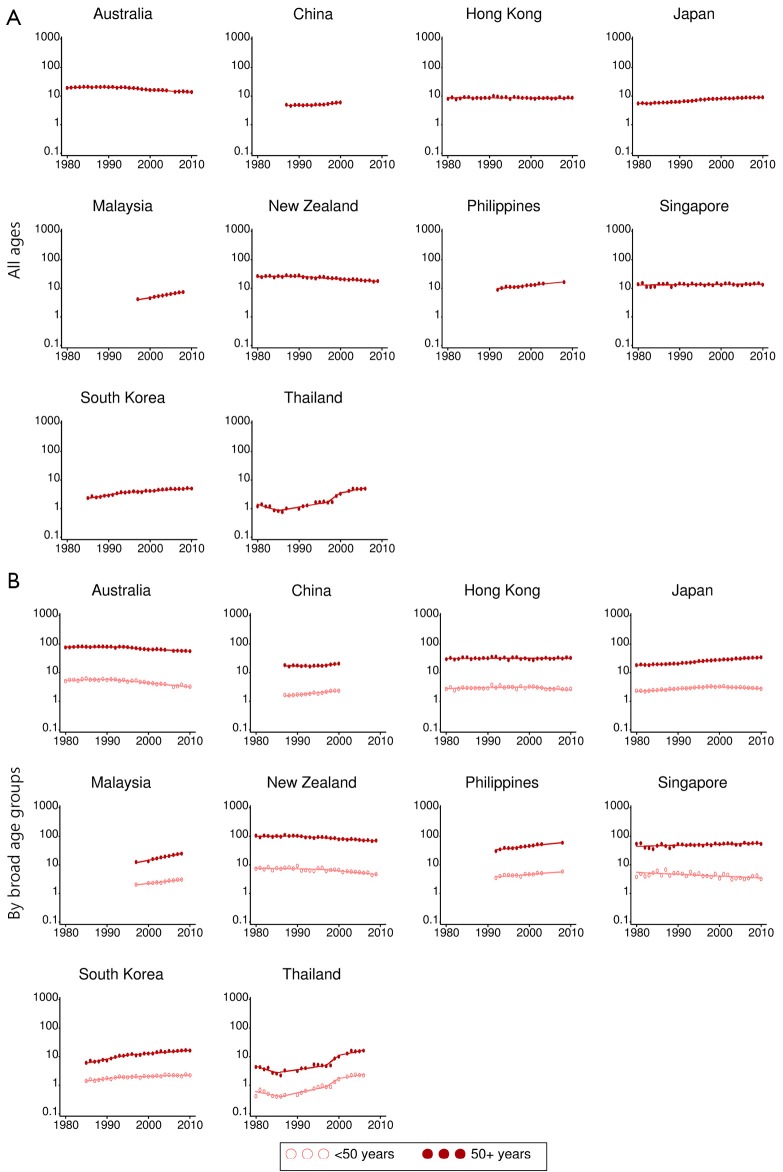
Breast cancer mortality rate trends for selected Asia-Pacific countries, 1980-2011. Notes: Y-axis is shown on a log scale. Rates were age-standardised to the World Standard Population^17^, and expressed per 100,000 female population. Years available differ by country. Data source: World Health Organization^18^.

Breast cancer mortality has been decreasing by an average of about 2% per year for females of all ages in Australia (2000-2011), and New Zealand (1989-2009), while the corresponding trends were stable for Hong Kong and Singapore (**Table 3** and **Figure 4**). In contrast, overall breast cancer mortality rates have increased in several other countries, with the largest rises recorded in Malaysia (6% per year between 1997-2008) and Thailand (7% per year between 2000-2006, with an average annual increase of 9% from 1985 onwards).

Significant differences were found in breast cancer mortality trends by age group in eight of the ten countries for which trend data were available. The exceptions were China and Thailand, where mortality rates were increasing rapidly irrespective of the age at death. In Australia and New Zealand there was a larger decrease in mortality rates for females under 50 years of age compared to those 50 years or older; in Hong Kong, a significant decrease was recorded in the under 50 age group while mortality rates were stable for females 50 years of age and over; in Japan and Singapore, breast cancer mortality was decreasing for younger females but increasing in the older age group; and in Malaysia, the Philippines and South Korea, although rates were increasing in both age groups, the rate of increase was lower for females under 50 years of age.

### Mortality to incidence rate ratio

The value of MR:IR for breast cancer was slightly lower in the Asia-Pacific region compared to the world average (0.27 and 0.30 respectively, **Table 1**). Within the region, MR:IR was lowest in Oceania (0.20) and highest in South-Eastern Asia (0.41). Country-specific estimates for MR:IR ranged from 0.12 in South Korea and 0.16 in Australia to 0.51 in Myanmar and the Solomon Islands and 0.53 in Papua New Guinea (**Table 1** and **Figure 1**).

## Discussion

### Incidence

The greatest increases in female breast cancer in the Asia-Pacific region are occurring in countries that, until recently, have had relatively low incidence rates. Our findings are consistent with other research that has shown moderate to rapid increases in breast cancer incidence in several countries throughout Eastern and South-Eastern Asia over the last two decades^31^^-^^34^. An analysis of breast cancer data from 15 population-based registries in China, Japan, the Philippines, Singapore, South Korea, Taiwan and Thailand^31^ found that in areas where the incidence rate was changing quickly the increases were occurring in all age groups, while increases were only happening for women aged 50 years and older in areas where there was a more modest trend. Even Mongolia, with the lowest estimated rate of female breast cancer in the Asia-Pacific region, was reported to be experiencing a gradual increase in incidence between 1998 and 2005^35^.

Two main differences are apparent when comparing the descriptive epidemiology of breast cancer in Asia to the West^36^. Incidence rates tend to be much lower for females in Asia (although the gap is decreasing). The other striking feature is the distribution in age at diagnosis, with the peak being in the 45-50 age range within many Asian countries, while a median of 55-60 years old is typical in most Western countries^36^^-^^38^. Even so, the age-specific incidence rates of breast cancer are still considerably higher in Australia and New Zealand compared to Asian countries among women younger than 50. It is generally accepted that the risk factors for breast cancer are similar throughout the world, even though most of the aetiological research has been conducted on Western populations^2^. However, the differences highlighted above suggest that the prevalence and composition of risk factors varies between the countries.

It is possible that the higher median age at diagnosis among women in Western countries could be partly explained by the population-based mammography breast screening that is widely available in these countries, which typically targets women aged 50 years and over^5^. One criticism of these programs is that asymptomatic breast tumours that would not progress further are over-diagnosed^5^, which has the potential to artificially inflate the incidence rate among women over 50 years old, and hence increase the observed median age at diagnosis.

However, it seems likely that most of the age difference is real. At least some of the tendency towards a younger age of onset for female breast cancer in Asia compared to Western countries such as Australia and New Zealand can be attributed to differences in life expectancy, with a greater proportion of the population in the younger age groups for females in developing countries^7^. Researchers have also identified a strong birth cohort effect^39^^,^^40^ that is reducing over time due to cultural changes. A large portion of the increase in breast cancer incidence being observed in many Asia-Pacific countries is likely due to the adoption of a more “Westernised” lifestyle, including adverse changes to diet, physical activity and fertility^31^^,^^41^^-^^44^. This effect has been greatest among younger women living in urban areas of lower and middle income countries^9^.

Reproductive issues that impact on lifetime exposure to estrogen have a particularly crucial role in the potential for development of female breast cancer^45^^,^^46^. Numerous case-control studies have established that the main risk factors for breast cancer in Asian women include early menarche, late menopause, older age at first delivery, and a lower number of full term pregnancies^47^^-^^51^. The prevalence of these reproductive risk factors is on the rise in Asia^44^. For example, family planning initiatives have brought about sustained declines in fertility rates across the region over recent decades^52^^,^^53^.

Genetic interactions may also alter the importance of some causal factors between ethnic groups^2^^,^^54^. Significant differences in age-specific incidence rates between Malay, Chinese and Indian women living in Singapore have been attributed to variations in the association between childbirth and pre-menopausal breast cancer by ethnicity^55^^,^^56^.

Other issues that may influence reported differences in breast cancer incidence between countries include socio-economic status, utilisation of mammography and the scope and accuracy of cancer registry data^57^. Higher rates of breast cancer are generally associated greater personal wealth, ease of access to breast cancer screening and areas where there are mechanisms in place for full population-based collection of all cases of cancer.

One of the consequences of the disparity in the average age at diagnosis between more developed and less developed countries is a marked difference in the distribution of tumour types; a greater proportion of cases in Asia are currently estrogen or progesterone receptor negative (ER–/PR–)^9^^,^^36^. However, the distribution appears to be changing in some countries. A study in Malaysia^58^ showed that between 1994 and 2008, the proportion of ER+ breast cancers increased by 2% for every 5 years cohort. This may be due to an increase in the incidence of ER+ cases while the incidence of ER– cases remained fairly stable, giving rise to a higher proportion of ER+ cancers over time.

It is therefore expected that the incidence rate, median age and biological profile of breast cancers throughout the Asia-Pacific will eventually come to more closely resemble that of Australia and New Zealand as changes to lifestyle and screening practices become more widespread, combined with a shift towards an older population structure^31^^,^^36^^-^^38^^,^^58^.

### Mortality

Mortality trends varied across the region, with large increases in several Asian countries contrasting with decreases in both Australia and New Zealand. A 3-fold increase in the breast cancer mortality rate has been predicted in South Korea between 1983 and 2020 if current patterns continue^59^. Trends can also vary within a country. Guo *et al*.^60^ reported a 20% increase in breast cancer mortality rates in rural parts of China compared to a decrease of 7% in urban areas between 1997-2001 and 2007-2009, although mortality rates still remained higher in urban areas.

Interesting results emerged for mortality trends by age. We found that in most countries, mortality from female breast cancer was either decreasing more quickly, or alternatively, increasing at a slower pace, for females under 50 years of age compared to those who were over 50. This was most noticeable in Japan and Singapore where there was a significant decrease in mortality for younger females compared to a significant increase in the breast cancer death rate among those who were older.

Survival from breast cancer depends mainly on early detection and access to optimal treatment. There are several cultural and economic obstacles involved in managing breast cancer in parts of the Asia-Pacific region, including misunderstanding about the disease (such as the incorrect idea that surgery will cause cancer cells to spread more quickly), geographical isolation, lack of education and awareness, inadequate diagnostic equipment and treatment facilities, competing health care needs and a reliance on traditional remedies^1^^,^^9^^,^^37^. These factors may influence treatment decisions and adherence^2^. Social implications, such as the possibility of abandonment following a mastectomy or the perception that a breast cancer patient may become a burden to her family^37^^,^^61^, can also cause fear, denial and reluctance to visit a doctor.

Delayed presentation is a major problem that stems from these barriers, with a high proportion of women with breast cancer in less developed Asian countries being diagnosed with advanced disease^1^^,^^2^^,^^37^. Tumours tend to be large with poorer histological grade, and many have lymph node involvement or distant metastases^1^^,^^62^^,^^63^.

Access to mammography is limited in many developing countries^9^. Hence, the majority of female breast cancer cases in most parts of the Asia-Pacific region are only detected after symptoms appear^1^. It is uncertain whether mammography screening would be as effective for women in Asia as it is for Caucasians, due to the tendency for Asians to have smaller volume breasts with denser tissue, even among post-menopausal women^2^^,^^64^. Studies have, however, shown that screening by mammography is superior to clinical examination for detecting breast cancer early among Japanese women^64^, and research in Singapore found that the spectrum of mammographic abnormalities observed was similar to what would be expected in a Caucasian population^65^. Apart from the physiological differences, there are also other issues to consider in regard to the use of mammography. Asian women have been reported to under-utilise breast cancer screening due to a variety of reasons such as a lack of knowledge, concerns about modesty, and the need for more encouragement from family and physicians^64^^,^^66^^,^^67^. Given the limited resources in many less developed areas, it has been suggested that, as a first step, available money would be better spent on improving breast cancer awareness through community-based education programs, teaching self-examination and encouraging women to obtain medical assistance for diagnosis and treatment^68^^,^^69^.

Ideal management of breast cancer requires a multidisciplinary team, comprising the breast surgeon, radiologist, pathologist, radiation and medical oncologists, plastic surgeon, and a breast care nurse specialist. It also depends on a robust and equitable health care system, with adequate staffing and resources to provide optimal treatment. Low and middle income countries form the majority of the Asia-Pacific region, where the proportion of government spending on health care is inadequate. Total expenditure on health as a percentage of the Gross Domestic Product is 5% or lower in many parts of the Asia-Pacific region compared to 9% in Australia and Japan and 10% in New Zealand^70^. The lower priority given to health expenditure in some countries, particularly for a female-specific disease such as breast cancer, means that even women who seek early medical intervention often do not receive appropriate advice or treatment^1^.

Tumour biology and ethnicity play a smaller role in breast cancer survival. There are racial differences in histological types of breast cancer as well as the molecular profile of breast cancer^71^. Triple negative breast cancer, where the tumour has an absence of oestrogen and progesterone receptors and no overexpression of human epidermal growth factor, has a poorer prognosis compared to other molecular subtypes^72^. Reports indicate that this type of breast cancer is relatively more common in the Asia-Pacific region compared to North America or Europe^9^. Researchers in Sarawak, Malaysia, showed that the triple negative subtype accounted for a higher proportion of breast cancer cases in Sarawak natives (37%) and Malay women (33%) compared to those of Chinese ethnicity (23%)^73^. Another study from Malaysia and Singapore found an independent effect of ethnicity on survival following female breast cancer, with poorer survival for Malay women compared to their Chinese and Indian counterparts^74^. In particular, Malay ethnicity was associated with more aggressive tumour biology as evidenced by a higher risk of axillary lymph node metastasis for tumours of a similar size.

Other reasons for survival disparities by ethnicity are yet to be fully determined, but may consist of a combination of socio-economic status, cultural factors, response to treatment and differences in lifestyle^74^. For example, treatments such as hormonal therapy and cytotoxic chemotherapy that work well for Caucasian women may not be as effective in Asia due to differences in tumour biology and metabolism of drugs^2^^,^^38^.

A lot of work has been done over the last decade to establish appropriate strategies for dealing with female breast cancer in developing countries with the aim of improving outcomes. The Breast Health Global Initiative was set up in 2003 by the Fred Hutchinson Cancer Centre in Seattle to develop economically feasible and culturally sensitive guidelines for breast cancer care in low and middle income countries^75^^,^^76^. These guidelines cover the whole spectrum of breast cancer control (prevention, early detection, diagnosis and treatment). Given the differentials in available funding, it would not be practical for low income countries to provide breast cancer care at the same level as high income countries. Therefore, the guidelines are stratified into basic, limited, enhanced and maximal depending on the resources that are available^37^^,^^77^. The aim of this stratification model is to ensure that even in low resource settings, women with breast cancer are managed appropriately.

Encouraging signs are starting to appear. Recent improvements in the survival rate of breast cancer patients have been reported in South Korea^33^ and Malaysia^78^. The mortality trends that we have reported here for women under 50 years of age would also seem to indicate that the message regarding the importance of early detection may be gaining traction among younger females throughout parts of the Asia-Pacific region. However, the full impact of programs such as the Breast Health Global Initiative will remain difficult to measure due to the lack of national or regional cancer data collections in many less developed countries^79^.

### Limitations

Our reporting of trends was limited in that data over time were only available from a relatively small number of countries/registries. Consequently, gaps remain in the description of how the incidence and mortality of female breast cancer is changing throughout the entire Asia-Pacific region.

We also did not report survival data mainly due to a lack of recent, reliable and comparable information from less developed countries, but have instead provided data on MR:IR as a proxy measure. Some of the higher values for MR:IR may be biased upwards as they could possibly represent better recording of mortality data compared to breast cancer incidence in those countries.

## Conclusion

Although breast cancer incidence rates currently remain lower in developing countries of the Asia-Pacific compared to Australia or New Zealand, the rapid increases that are being experienced in places with large populations such as China and Japan will continue to shift the worldwide burden of this disease towards Asia^80^. Female breast cancer must therefore be afforded a higher priority for health spending in the region. While opportunities exist for worldwide collaborations to improve outcomes for women with breast cancer, at the same time it is clear that the approach to breast cancer control needs to be evaluated and tailored to the different situations in each country^68^^,^^77^^,^^81^. More emphasis also needs to be placed on cancer prevention strategies and the development of population-based registration systems for the effective planning and monitoring of cancer control programs^82^^,^^83^. The ultimate goal is to ensure that the declines in breast cancer mortality rates seen in Australia and New Zealand are replicated in other countries throughout the Asia-Pacific region in the near future.
